# Supplementary material for: Digital mental health service engagement changes during Covid-19 in children and young people across the UK: Presenting concerns, service activity, and access by gender, ethnicity, and deprivation
Source: PLoS One. 2025 Feb 13;20(2):e0316468. doi: 10.1371/journal.pone.0316468 (PMC11825017; doi:10.1371/journal.pone.0316468)
Supplement: S1 Table — a. The proportion of research consent responses relative to gender in the digital service. b. The proportion of research consent response relative to ethnicity in the digital service. (ZIP) [file pone.0316468.s001.zip › S1a_Table.pdf]

Supplementary Materials:

Table S1a. The proportion of research consent response relative to gender in the digital service.

	No consented (%)	Consented (%)
Agender	43.87	56.13
Female	43.24	56.76
Gender Fluid	42.66	57.34
Male	38.89	61.11
Total	42.29	57.71
