# Supplementary material for: Digital mental health service engagement changes during Covid-19 in children and young people across the UK: Presenting concerns, service activity, and access by gender, ethnicity, and deprivation
Source: PLoS One. 2025 Feb 13;20(2):e0316468. doi: 10.1371/journal.pone.0316468 (PMC11825017; doi:10.1371/journal.pone.0316468)
Supplement: S2 Table — (PDF) [file pone.0316468.s002.pdf]

Table S2. Presenting concerns frequency and categorization for the study based on previous literature on Covid-19 mental health impact studies.

Studies	Codes	Categories[1]	Presenting concerns	Frequency
Son, Hedge, Smith, Wang & Sasangohar., 2020[29]	External	School or home related issue	Family Relationships	11250
	External	School or home related issue	Friendships	9272
	External	School or home related issue	School/College issues	4741
	External	School or home related issue	Bullying	4636
	External	School or home related issue	Bereavement	3547
	MH_2	Mental health/emotional difficulties	Anxiety/Stress	24036
	MH_2	Mental health/emotional difficulties	Self-Worth	6495
	MH_2	Mental health/emotional difficulties	Depression	6394
	MH_2	Mental health/emotional difficulties	Eating Difficulties	4095
	MH_2	Mental health/emotional difficulties	Sadness	3989
Ford, John & Gunnell., 2021[30]	PhyOth	Physical or other issue	Illness (own)	1037
	PhyOth	Physical or other issue	Weight Issues	723
	PhyOth	Physical or other issue	Illness (other)	720
	PhyOth	Physical or other issue	Access to resources	655
	PhyOth	Physical or other issue	Teenage Pregnancy	351
Taggart, Rouf, Hisham, Duckworth & Sweeney, 2021[31]	Rsk_2	Risk / Abuse /Safeguarding risk	Trauma	1672
	Rsk_2	Risk / Abuse /Safeguarding risk	Sexual Abuse	1297
	Rsk_2	Risk / Abuse /Safeguarding risk	CSE	887
	Rsk_2	Risk / Abuse /Safeguarding risk	Emotional Abuse	724
	Rsk_2	Risk / Abuse /Safeguarding risk	Physical Abuse	568
Taquet, Luciano, Geddes & Harrison, 2021[32] Findaly & Gilmore, 2020[33]	SSMal_2	Suicidality / Self Harm / Maladaptive coping	Self-Harm	12185
	SSMal_2	Suicidality / Self Harm / Maladaptive coping	Suicidal Thoughts	10776
	SSMal_2	Suicidality / Self Harm / Maladaptive coping	Substance Misuse	478
	SSMal_2	Suicidality / Self Harm / Maladaptive coping	Alcohol (self)	340
	SSMal_2	Suicidality / Self Harm / Maladaptive coping	Criminal Behaviour	109

[1] Tables shows the top 5 presenting concerns in each category only
