# Supplementary material for: Digital mental health service engagement changes during Covid-19 in children and young people across the UK: Presenting concerns, service activity, and access by gender, ethnicity, and deprivation
Source: PLoS One. 2025 Feb 13;20(2):e0316468. doi: 10.1371/journal.pone.0316468 (PMC11825017; doi:10.1371/journal.pone.0316468)
Supplement: Attachment — Submitted filename: Response to Reviewers_ PLOS ONE_ Covid-19 Kooth .docx [file pone.0316468.s003.docx]

12th September 2024

Dear Giulia Ballarotto

Thank you for your assistance and review of the manuscript *“Digital Mental Health Service engagement changes during Covid-19 in children and young people across the UK: presenting concerns, service activity, and access by gender, ethnicity, and deprivation”.*

I am pleased to return the manuscript after major revisions, which address the reviewer's comments. We now feel that the manuscript is of higher quality and will fit the scope of PLOS ONE well. We would like to thank the editors for the flexibility in resubmission due to the maternity leave of our first author.

We have added, as suggested, a revised Competing Interests section here as well as in the mauscript;

*“We note that one or more of the authors are employed by a commercial company: Kooth Digital Health. This does not alter our adherence to PLOS ONE policies on sharing data and materials.*

*Dr Louisa Salhi, Dr Nimrah Afzal, Samaryah Sammut and Aaron Sefi are currently employed by Kooth Digital Health and receive honorarium for their time. Dr Louisa Salhi also holds honorary researcher status at the University of Kent and the University of Manchester. Dr Nimrah Afzal holds an honorary research position at the University of Bath.*

*Dr Santiago de Ossorno Gardia and Lily Mainstone-Cotton were previously employed by Kooth Digital Health at the time of data extraction and received honorarium for their time, but are no longer employed by Kooth Digital Health.*

*Duuleka Knipe, Amanda Marchant and Ann John declare no conflict of interest, no remuneration was received from this work.”*

We confirm that neither the manuscript nor any parts of its content are currently under consideration or published in another journal. All authors have approved the manuscript and agree with its revised submission to the PLOS ONE. For ease of reviewing our changes, we have provided direct responses to the reviewers below this cover letter as well as a marked-up manuscript copy and a clean copy.

Best Regards,

Dr Louisa Salhi (Corresponding author)

1. Please ensure that your manuscript meets PLOS ONE's style requirements, including those for file naming. The PLOS ONE style templates can be found at

https://journals.plos.org/plosone/s/file?id=wjVg/PLOSOne_formatting_sample_main_body.pdf and
<https://journals.plos.org/plosone/s/file?id=ba62/PLOSOne_formatting_sample_title_authors_affiliations.pdf>

Thank you for highlighting this, we have since changed these.

2. Thank you for stating the following in the Competing Interests section:

I have read the journal's policy and the authors of this manuscript have the following competing interests:

“*Dr Louisa Salhi, Dr Nimrah Afzal, Samaryah Sammut and Aaron Sefi are currently employed by Kooth Digital Health - the service that the service data was extracted from. Dr Louisa Salhi also holds honorary researcher status at the University of Kent and the University of Manchester. Dr Nimrah Afzal holds an honorary research position at the University of Bath.*

*Dr Santiago de Ossorno Gardia and Lily Mainstone-Cotton were previously employed by Kooth Digital Health at the time of data extraction but are no longer employed by Kooth Digital Health.*

*All other authors have no conflict of interest.*

*We note that one or more of the authors are employed by a commercial company: Kooth Digital Health. This does not alter our adherence to PLOS ONE policies on sharing data and materials.*”

Thank you for raising this - we have now clarified this in the manuscript and highlighted the changes above in red.

(1) Please provide an amended Funding Statement declaring this commercial affiliation, as well as a statement regarding the Role of Funders in your study. If the funding organization did not play a role in the study design, data collection and analysis, decision to publish, or preparation of the manuscript and only provided financial support in the form of authors' salaries and/or research materials, please review your statements relating to the author contributions, and ensure you have specifically and accurately indicated the role(s) that these authors had in your study. You can update author roles in the Author Contributions section of the online submission form.

Please also include the following statement within your amended Funding Statement.
“The funder provided support in the form of salaries for authors [insert relevant initials], but did not have any additional role in the study design, data collection and analysis, decision to publish, or preparation of the manuscript. The specific roles of these authors are articulated in the ‘author contributions’ section.”
If your commercial affiliation did play a role in your study, please state and explain this role within your updated Funding Statement.

Thank you - we have now made this clearer in the manuscript.

(2) Please also provide an updated Competing Interests Statement declaring this commercial affiliation along with any other relevant declarations relating to employment, consultancy, patents, products in development, or marketed products, etc.

Within your Competing Interests Statement, please confirm that this commercial affiliation does not alter your adherence to all PLOS ONE policies on sharing data and materials by including the following statement: ""This does not alter our adherence to PLOS ONE policies on sharing data and materials.” (as detailed online in our guide for authors http://journals.plos.org/plosone/s/competing-interests) . If this adherence statement is not accurate and there are restrictions on sharing of data and/or materials, please state these. Please note that we cannot proceed with consideration of your article until this information has been declared.

Please include both an updated Funding Statement and Competing Interests Statement in your cover letter. We will change the online submission form on your behalf.

We have added this to the above section along with our Competing Interests and added it to the above cover letter.

3. We note that you have indicated that there are restrictions to data sharing for this study. For studies involving human research participant data or other sensitive data, we encourage authors to share de-identified or anonymized data. However, when data cannot be publicly shared for ethical reasons, we allow authors to make their data sets available upon request. For information on unacceptable data access restrictions, please see http://journals.plos.org/plosone/s/data-availability#loc-unacceptable-data-access-restrictions.

Before we proceed with your manuscript, please address the following prompts:

a) If there are ethical or legal restrictions on sharing a de-identified data set, please explain them in detail (e.g., data contain potentially identifying or sensitive patient information, data are owned by a third-party organization, etc.) and who has imposed them (e.g., a Research Ethics Committee or Institutional Review Board, etc.). Please also provide contact information for a data access committee, ethics committee, or other institutional body to which data requests may be sent.

b) If there are no restrictions, please upload the minimal anonymized data set necessary to replicate your study findings to a stable, public repository and provide us with the relevant URLs, DOIs, or accession numbers. Please see http://www.bmj.com/content/340/bmj.c181.long for guidelines on how to de-identify and prepare clinical data for publication. For a list of recommended repositories, please see https://journals.plos.org/plosone/s/recommended-repositories. You also have the option of uploading the data as Supporting Information files, but we would recommend depositing data directly to a data repository if possible.

Please update your Data Availability statement in the submission form accordingly.

We are not able to share the data in this instance due to the privacy policies set out by the services.

“The data analysed in this study is subject to the following licenses/ restrictions: Under Kooth’s privacy policy, data can only be shared with trusted partners for research studies dedicated to improving the service. Requests to access these datasets should be directed to the research department at Kooth; [research@kooth.com](mailto:research@kooth.com), where the author LS is head of the department.”

4. When completing the data availability statement of the submission form, you indicated that you will make your data available on acceptance. We strongly recommend all authors decide on a data sharing plan before acceptance, as the process can be lengthy and hold up publication timelines. Please note that, though access restrictions are acceptable now, your entire data will need to be made freely accessible if your manuscript is accepted for publication. This policy applies to all data except where public deposition would breach compliance with the protocol approved by your research ethics board. If you are unable to adhere to our open data policy, please kindly revise your statement to explain your reasoning and we will seek the editor's input on an exemption. Please be assured that, once you have provided your new statement, the assessment of your exemption will not hold up the peer review process.

We are not able to make the data openly available but have now added contact details in order for data access requests to be sent to.

5. Your ethics statement should only appear in the Methods section of your manuscript. If your ethics statement is written in any section besides the Methods, please delete it from any other section.

Thank you - we have amended this in the manuscript.

6. We note you have included a table to which you do not refer in the text of your manuscript. Please ensure that you refer to Table 4 in your text; if accepted, production will need this reference to link the reader to the Table.

Thank you for identifying this - we have now amended this in the text.

7. Please include captions for your Supporting Information files at the end of your manuscript, and update any in-text citations to match accordingly. Please see our Supporting Information guidelines for more information: <http://journals.plos.org/plosone/s/supporting-information>.

Review this

**Additional Editor Comments:**

I have seen the paper and the suggestions from the reviewers. In particular, I urge the authors to pay attention to the indications provided by Reviewer 2, who highlighted several critical points. Additionally, I encourage the authors to utilize a proofreading service, given the numerous errors in the text and the usage of the English language.

Thank you for this review and the comments. We have addressed all the comments suggested.

## Reviewers' comments:

**Comments to the Author**

1. Is the manuscript technically sound, and do the data support the conclusions?

The manuscript must describe a technically sound piece of scientific research with data that supports the conclusions. Experiments must have been conducted rigorously, with appropriate controls, replication, and sample sizes. The conclusions must be drawn appropriately based on the data presented.

Reviewer #1: Yes

Reviewer #2: Partly

2. Has the statistical analysis been performed appropriately and rigorously?

Reviewer #1: Yes

Reviewer #2: Yes

3. Have the authors made all data underlying the findings in their manuscript fully available?

The [PLOS Data policy](http://www.plosone.org/static/policies.action#sharing) requires authors to make all data underlying the findings described in their manuscript fully available without restriction, with rare exception (please refer to the Data Availability Statement in the manuscript PDF file). The data should be provided as part of the manuscript or its supporting information, or deposited to a public repository. For example, in addition to summary statistics, the data points behind means, medians and variance measures should be available. If there are restrictions on publicly sharing data—e.g. participant privacy or use of data from a third party—those must be specified.

Reviewer #1: No

Reviewer #2: No

4. Is the manuscript presented in an intelligible fashion and written in standard English?

PLOS ONE does not copyedit accepted manuscripts, so the language in submitted articles must be clear, correct, and unambiguous. Any typographical or grammatical errors should be corrected at revision, so please note any specific errors here.

Reviewer #1: No

Reviewer #2: No

We have since reviewed this and improved the manuscript’s readability, grammar and spelling. We regret that these mistakes were presented in the first submission.

**Review Comments to the Author**

Please use the space provided to explain your answers to the questions above. You may also include additional comments for the author, including concerns about dual publication, research ethics, or publication ethics. (Please upload your review as an attachment if it exceeds 20,000 characters)

**Reviewer #1:** This is a potentially valuable descriptive study addressing a topic of high policy priority. The study appears to have been appropriately conducted, but the write up needs a significant overhaul. There are multiple errors throughout the manuscript that suggest inadequate attention to copy editing prior to submission. These errors take the form of the inclusion of legacy placeholder text, incorrect referencing of a table/figure, nonsensical sentences and a disconnect between the number of studies referred to (multiple) and supporting citations (singular). Other potential improvements include: framing this more explicitly as an evaluation of changes observed in one digital mental health service rather than digital mental health services more generally (eg modify title, describe role of Kooth in broader ecosystem, discuss generalisability to digital mental health services more widely); shorten discussion and focus on both lessons for Kooth and UK/international digital mental health services; and add nuance to statements about attractiveness of Kooth type services by referencing the youth and digital mental health services preference literature.

Thank you for this comment and review, we have now made substantial revisions to improve the readability, flow as well as framing in the introduction. We have also improved the discussion in light of your suggestions.

**Reviewer #2:** This study investigates CYP service use before and during the COVID pandemic. It stratifies its findings by type of use, reason for accessing service and demographic variables. Overall service use increased rapidly at pandemic inception but then reduced, though remained higher than pre-pandemic levels, time-series analysis revealed overall small reduction in rate of change of use during pandemic.

1) Introduction
Can the intro better relate to the research questions more clearly, why is referral route an important research question to ask? How does RQ about presenting concerns relate to the first paragraph.
“. Some countries responded with a national digital support provision for mental health in the face of the pandemic 10, but it is not yet clear how well these services were utilized, or whether access was equitable across sociodemographic groups.”

Is Kooth a UK “national digital support provision” - if so, probably should state in the introduction and explain a little bit about how CYP can access this service to orient the reader.

The introduction has been updated thoroughly and shown with the markdown copy in red (Pages 1- 3)

The introduction was rewritten to address the comments. Additional sources of literature were included to add more context and further support existing statements, such as in explaining the relationship between particular demographics and mental health during the COVID-19 pandemic. More context and rationale were added to support the research questions that are presented in the intro. Changes in trends that have been tied to COVID-19 pandemic lockdowns and the related presenting issues were explained. Context was added in relation to the Kooth platform and service.

2) Methods
Outcome variable
“Service access contacts was our outcome of interest by month. To calculate the rate of contact per user, we identified the number of active users on the platform (i.e., denominator) by counting the number of users accessing the service at least once each month between June 2019 to April 2021.”
For clarity can you state that the numerator was the number of contacts, denominator number of active users providing a rate of contacts/user.

This has now been updated in Section 2.3 (Page 4)

3) CCGs:
“A total of 34 regions structured by Clinical Commissioning Groups (CCG) with unchanged resource contracts were selected from a total of 97 (N=5 decreased; N=58 increased); the selection criteria of regions was used to prevent biases due to changes in resources that may affect the demand and capacity of the online service during the pandemic (resource increase or decrease during the observational period). The study selected only regions of the service that had the same allocated number of resources before and during the pandemic, remaining therefore constant during the period.”
You need to explain what a CCG is and how it fits into the UK healthcare system to a non-UK audience or any reader who is unaware of how services are commissioned in the UK.

What do you mean by resource allocation – resource allocated to Kooth, resource allocated to the CCG?
How did you know resource allocation had increased decreased? please state and cite where this information was obtained if relevant.
This will then make it clear why you selected only 34/97 CCGs, which shall reduce the number of users included in the analysis.

Thank you for this comment. We have amended the wording in section 2.2. It was only relevant to examine data to see changes due to COVID-19 pandemic and the first lockdown in the UK where contracts were stable and not going through a commercially driven uplift or reduction. This was done to prevent bias, for example in contracts where there was an uplift in contract reach, there would likely be an increase in utilisation. Therefore confounding our results.

Pages 3-4 to clarify.

4) IMD
Typically, this is known as the index of multiple deprivation, no deprivations.

This has been corrected in the manuscript.

5) Can you clarify that no part of the user postcode was used to determine the IMD.

This information has been added to section 2.2 (Pages 3-4). The service does not store postcode data of any kind and so this was not used to determine IMD.

6) Can you clarify what you mean by “partially disclosed” when you state “We used the average IMD rank of each CCG location included (out of a possible 191(ranks?)) to calculate deprivation quintiles (higher ranks are least deprived) for each user in relevance to their area ~~(partially disclosed at registration and group by CCG)~~.”

We have removed ‘partially disclosed’ as this was misleading and confusing to the reader.

7) Why are 29.4% of IMD data missing for CCGs, given this information is publicly available?

The incompleteness is due to imperfect matching between the contract areas and the CCG areas as Kooth can be commissioned for partial areas as well as whole CCG areas. Contracts commissioned by the NHS in Kooth are not always a perfect match to CCG areas and could be commissioned through trusts or health boards for example.

We have updated the methods section 2.2 (Pages 4) to clarify this.

8) Gender
Can you describe for the reader what genders are included in the terms agender and gender fluid.

This reflected the options available to choose from at the time. We have added more detail to the section has been updated (Page 4).

9) Presenting concerns
“A total of 118 reported different presenting concerns were identified for the study. Those were grouped into five high-level categories aligned with previous literature on the mental health impact of the pandemic and allowed for sufficient observations within each category to track trends over time.”
This information is presented later on
“The dataset presented 118 different types of presenting concerns, which were grouped into five categories representing the main mental health impacts of the Covid-19 pandemic for CYP identified in previous literature (Supplementary Table 2).”
Can you present this information only once and state what the categories are, which authors grouped them and how disagreements were resolved.

This information has been removed from Page 4 and the section (Section 2.2) has been updated to read;

10) Analysis
“For this, we included a binary coded variable in the model which represented the pandemic period (i.e., model step change), as well as an interaction term between time and intervention which models a slope change. “
In the interaction term, what was the reference period?

Section 2.4 (Pages 4-5) has been updated to include this information;

11) General point:
Could the time Kooth commenced in a CCG affect rate of change pre-pandemic, for example rate of change might be higher upon commencement and then stabilise.

Thank you for raising this point. We have added some discussion of this to the limitations section 4.5 (Page 15)

12) Results
The study would benefit from a description of how the sample were selected, the number excluded from CCG selection aswell as the number excluded from lack of consent and any other exclusion criteria. Can you also state the proportion of registered users who transferred to active users and therefore included in the analysis (some may register but never contact the service directly or indirectly).

We describe the process of CCG selection in the methods section and additional detail has been added to this (see response to comments above). Data on the total number of Kooth users was not deemed necessary for the purpose of this study and as such was not included in the data extract. Similarly we have not included counts of non-consenting individuals. However, we have included exploration of demographic differences between consenting and non-consenting individuals (Supplementary Table 1) which the service provided on an aggregate level. An additional demographic table has also been added (see below) as well as further details to the results section 3.1 (Page 5).

13) We are also missing N’s, would help interpretation of the table in tables 3, 4 and 5 confidence intervals. For example in table 5 small Ns in Q2, Q3 and Q4 are indicated by wide intervals.

An additional table has been added to section 3.2 (Pages 5-8) to add this additional detail.

Table 1 Descriptive table of users and contacts pre pandemic and during pandemic

|  |  | COVID period* | |
| --- | --- | --- | --- |
|  |  | Pre- | During |
| **Users** | |  |  |
| Mean no. of active users per month (SD) | | 3579 (572.9) | 3792 (724.6) |
| Mean no. of active users per month by deprivation quintile (SD) | |  |  |
|  | Q1 - most deprived | 820 (117.6) | 864 (173.8) |
|  | Q2 | 551 (109.6) | 506 (83.7) |
|  | Q3 | 468 (87.3) | 577 (166.9) |
|  | Q4 | 534 (168.4) | 555 (106.8) |
|  | Q5 - least deprived | 178 (51.3) | 148 (30.4) |
|  | Missing | 1029 (148) | 1143 (207.5) |
| **Contacts** | |  |  |
| No. of contacts | | 189011 | 88687 |
| Mean no. of contacts per month (SD) | | 7000 (1215.7) | 7391 (1243.9) |
| Number of contacts n(%) | |  |  |
|  | Article Comment | 6470 (3.4) | 1467 (1.7) |
|  | Chat | 29490 (15.6) | 12442 (14) |
|  | Forum Article Created | 9193 (4.9) | 5809 (6.6) |
|  | Forum Comment | 13658 (7.2) | 9786 (11) |
|  | Goal | 20652 (10.9) | 8957 (10.1) |
|  | Journal | 62163 (32.9) | 31411 (35.4) |
|  | Live Forum Comment | 1804 (1) | 400 (0.5) |
|  | Message Sent | 45581 (24.1) | 18415 (20.8) |
|  | Missing | 0 | 0 |
| Number of contacts by type n(%) | |  |  |
|  | Indirect | 75071 (39.7) | 30857 (34.8) |
|  | Direct | 113940 (60.3) | 57830 (65.2) |

* Pre-pandemic (1^st^ Jan 2018-31^st^ Mar 2020); During (1^st^ April 2020-31^st^ Mar 2021)

14) Supplementary Table 3 is missing from the supplementary.

Thank you for drawing our attention to this. We have removed the reference to Supplementary Table 3 from the text as Table 1 now sufficiently provides this information.

15) Main findings:
“When comparing trends in the periods, before the pandemic there was evidence of a 25% (95% CI: 9%- 44%) increase rate in contacts per user per month (p=.001), this reduced to a 21% (95% CI: 4%-41%) increase per month during the pandemic” – missing a fullstop.

This has been corrected.

16) “this reduced to a 21% (95% CI: 4%-41%) increase per month during the pandemic”
Where is this statement reflected in table 1 and figure 1?

Thank you for this comment. The 21% post-intervention slope can be calculated from the values in Table 2 (previously Table 1). We have added additional detail to the text to clarify this on (Page 6).

17) Overall, the figure shows a gradual increase in use of Kooth that sharply increases at the onset of the pandemic then gradually decreases, but does not return to pre-pandemic levels. The figure might benefit from a dashed line which shows what Kooth contacts would have looked like had the pre-pandemic slope continued. This may visually explain why the rate ratio of slope change in table 1 indicates a relative decrease in use (RR<1).

Thank you for this suggestion. While we would have liked to add this time restrictions and access to the data mean that we cannot make edits to this figure. We have instead added further detail to the text to assist in interpretation (Page 6) - please see response to previous comment above.

18) Similar questions about the same statistics for direct and indirect contacts.

This section has been reworked for clarity (Page 7).

19) Figure 2
Why in figure 2 do the pre-pandemic slopes by type of presenting concern indicate decreasing contacts per month, but in figure 1 when presented overall, indicate increasing contacts per month?
Are these sub-populations, if so this needs to be stated?
An explanation of external needs to be included in the main results and not just in the supplementary to aid the reader.

Thank you for this comment, we agree that further clarification is needed. Each contact may have multiple presenting concerns recorded. As such it is not a case of the overall contacts per user being broken into mutually exclusive categories or subgroups. We have updated the wording in the methods section (Pages 8-9) - please see response to previous comments regarding methods sections above.

The results section 3.4 (Pages 8-9) also adds further clarification to this point;

20) Needs to be reproduced with the X axis and the legend presented suitable for publication.

Replaced with updated figure - Figure 2 (Page 11).

21) From the figure it appears there is a slope change in self-harm/suicidality that was non-significant in the model, is it worth mentioning this?

We have added some additional detail to the text in section 3.4 (Pages 8-9).

‘Following this initial increase in contacts a non-significant downward trend was resumed for all presenting concerns with the exception of suicidality/self-harm/maladaptive coping where a slight upward trend can be seen, however this did not reach statistical significance.’

22) Again, lines to indicate what Kooth use would have looked like had the pre-pandemic slope continued may facilitate understanding of the table statistics.

Thank you for this comment, as above we are not able to go back to the original data to replot these lines. However, as previously we have updated the text for further interpretation in section 3.4 (Pages 8-9)

23) Discussion
Overall, this section needs to be rewritten prior to publication. The results need to be summarised more clearly and concisely. As do the main implications of the findings.

Thank you for highlighting how the discussion can be improved. This has been rewritten to ensure that the findings and their implications are clear and concise. (Pages 12-17).

24) Example: Very long and incomplete sentence. “We found that service activity changed and increase during the pandemic but changes in the referral sources and the way people access to pathways of care more generally may have impacted the increase in service access and demand, as contacts per users were higher in pre-pandemic activity when compared, our data suggest that dramatic changes in the healthcare system are likely to impact too digital ecosystems of support, even when operating anonymously and with relatively ease of access online, interoperability between services may be important in order to .” Please be more specific here and link your interpretation to the specific result your draw your inference from.

Thank you for drawing attention to this. This opening paragraph has been cut down to be more concise, and this sentence has been taken out. The opening paragraph provides a brief overview of key findings.

“For example on pages 12-13: *Overall, findings demonstrated a change in service use at the onset of the pandemic in March 2020. Specific findings included changes in the number of contacts with Kooth per user, certain referral sources and specific presenting issues, but more limited changes in regard to socio-demographics characteristics.”*

Interpretations of findings have been linked to specific results in the following sub-sections. For example, under ‘4.1. Changes in Referrals and Pathways to Receive Digital Care’ (Page 13)

*“In terms of referrals to Kooth, findings from the present analyses found a step change at the beginning of the pandemic period, with an increase in referrals via family/friends, care/social/charity, and social media/internet. Conversely, there was a significant decrease in referrals to Kooth via educational settings. These findings can be interpreted in light of lockdown restrictions meaning that CYP were not attending school (Timmins, 2021). Therefore, with schools closed, promotion of Kooth via schools was no longer possible.”*

25) Please refer to this paper Effects of the COVID-19 pandemic on primary care-recorded mental illness and self-harm episodes in the UK: a population-based cohort study (thelancet.com)
This examines trends in MI presentations by age and suggests that self-harm presentations were alarmingly low. Potentially the increased contacts seen in Kooth provided a resource for children and adolescents who were unable/unwilling to present to primary care. The paper also provides an example of how to visualise expected and observed trends, which the authors may find useful.

Thank you for suggesting this paper. This has been integrated into the interpretation of findings regarding user mental health and wellbeing presentations. For example:

*“A significant step-change increase was found for all except physical health during the first lockdown, with no evidence of a sustained increase following the pandemic lockdown. These findings are inconsistent with research suggesting that general practices in the UK observed a sharp decrease in CYP presenting with anxiety, depression, and self-harm (Carr et al., 2021). Despite this, findings from the present analyses of CYP presenting to Kooth and wider research do suggest immediate increases in mental health difficulties across young people and adults at the onset of the initial Covid-19 UK lockdown (Kwong et al., 2021; O’Connor et al., 2021).” (Page 14)*

26) I am not sure we can draw firm conclusions from the CCG IMD analysis about the facility of Kooth to reach deprived populations - each CCG represents 200 to 500K residents, pops are much larger in London CCGs, within those areas deprivation will vary widely, without a more localised measure of deprivation it is difficult to be certain that Kooth did truly reach these groups using the data in this study. However, this is noted in the limitations.

Thank you for highlighting this limitation of the CCG IMD analysis. This part of the discussion has been expanded, and the limitations have been highlighted even further. For example on page 16:

*“A significant step change increase was also observed for CYP from the highest quartile. There are several possible reasons for this. Firstly, research suggests that those from higher socioeconomic quartiles are more likely to have higher mental health literacy (Holman, 2015), which is linked to greater help-seeking (Steele et al., 2007; Zhang et al., 2023). Secondly, it is possible that CYP from higher socioeconomic backgrounds were more likely to already be in the mental healthcare system. Those from higher socioeconomic backgrounds are more likely to access mental health support (Morris et al., 2005) and are more likely to utilise private healthcare (Independent Healthcare Providers Network, 2023). As such these CYP may have been more likely to have been referred to Kooth by physicians.*

*However, these findings should be interpreted with caution because the CCG IMD represented 200,000 to 500,000 residents and did not provide a localised measure of deprivation. Thus, it is difficult to accurately ascertain the reach of Kooth across the different groups - particularly the lower quartiles. Nonetheless, these findings provide a brief insight into the importance of tailoring engagement strategies to demographics groups, and aiming to integrate into healthcare systems and communities at both a national and local level. Improving engagement strategies can ensure that user needs are met and access can be boosted and retained during and after times of need (Guest et al., 2023).“*

27) Do you have any hypotheses as to why Asian and mixed CYP did not increase use at pandemic onset?

Thank you for highlighting this. The interpretation of findings regarding user ethnicity has been expanded to explore specific differences between groups, including those from Asian and Mixed ethnic backgrounds. For example on page 15:

*“However, the differences in access between different ethnic minority groups paints a more detailed picture. Critically, the lack of significant increase among Asian/Asian British can be interpreted in light of existing research which suggests differences in mental health-related stigma across ethnic minority groups (Knifton, 2012). Furthermore, research has found that, compared with individuals from Black ethnic backgrounds, individuals from Asian backgrounds reported higher levels of self-stigma in relation to mental health (Misra et al., 2021). This could be due to different cultural norms and beliefs surrounding the conceptualisation of mental health and seeking support (Cheng et al., 2013).”*

28) In the final conclusion paragraph the authors state “Whilst the cause for the decrease in access as the pandemic continued is not known” – I’d really like them to suggest some possible hypotheses for why this might be: what about pandemic apathy/ of users, pandemic apathy of CYP settings to promote Kooth, increasing resilience of CYP as initial crisis passed, or bias towards larger numbers of less active users each month (those registered for long periods of time) as time progressed?

Thank you for highlighting this, and for suggesting a potential explanation for decrease in access as the pandemic continued. Instead of interpreting these findings in the discussion, this has been explored under sub-section ‘4.2 Changes in Service Access’ (Page 13)

*“It is noteworthy that the immediate increase in Kooth’s service access was followed by a rate of engagement which was lower than what was observed before the start of the pandemic. Two reasons could explain this. Firstly, it is possible that following lockdown, CYP adjusted to the situation, and potentially experienced some emotional stabilisation (Fancourt et al., 2021). Thus, there could be reduced demand for support. Conversely, this post-pandemic reduction also aligns with findings from other mental health services in the UK which also demonstrated a reduction of service utilisation following the Covid-19 pandemic (Gunnell et al., 2020; Tromans et al., 2020) . Furthermore, it is likely that there was an increase of demand for face-to-face mental health service post-lockdown, with figures suggesting some return to pre-pandemic activity (Chen et al., 2020).”*
