# Supplementary material for: Digital mental health service engagement changes during Covid-19 in children and young people across the UK: Presenting concerns, service activity, and access by gender, ethnicity, and deprivation
Source: PLoS One. 2025 Feb 13;20(2):e0316468. doi: 10.1371/journal.pone.0316468 (PMC11825017; doi:10.1371/journal.pone.0316468)
Supplement: Attachment — Submitted filename: Covid Reviewer Comments Nov 24.docx [file pone.0316468.s004.docx]

Dear Dr Goymer,

Thank you for your response and for sending the reviewer comments of the manuscript “*Digital Mental Health Service engagement changes during Covid-19 in children and young people across the UK: presenting concerns, service activity, and access by gender, ethnicity, and deprivation*”.

I am pleased to return the manuscript after minor revisions addressing the reviewer's comments. Please see the responses to the reviewer’s comments below.

1. I think more detail could be provided about the type of research this is and references to support the approach taken, i.e., for me, it is a clinical data mining (CDM) study. So, the work of Epstein for example could be cited (see: Epstein, I. (2009). Clinical data-mining: Integrating practice and research. Oxford University Press.). There are also other sources that may be suitable, e.g., Davenhill, R., & Patrick, M. (Eds.). (1998). Rethinking clinical audit: The case of psychotherapy services in the NHS. Psychology Press.

- Thank you for highlighting that the research approach could be emphasised. We have researched the clinical data mining approach and believe that this study does not fall under this approach, and neither is it a clinical data audit. This study is a hypothesis-driven time series analysis, which has been highlighted in the abstract, aims, and methods section. For example, in the aims on page 3: “*We aimed to assess the impact of the pandemic on service use in terms of overall contact, referral source, type of contact, and the type of presenting issue (e.g. mental health, abuse) using interrupted time-series modelling, and explored whether the impact is different between sociodemographic groups (gender, ethnicity and deprivation level) in one digital mental health service for CYP operating in the UK.”* and in the methods on page 4: “*An interrupted time-series analysis was used to assess whether there was evidence of a change in online service use during the pandemic period (April 2020 - April 2021) compared to pre-pandemic (June 2019 - March 2020) trends*.”
- However, we made some additions to further highlight the hypothesis-driven nature of this study in the **Abstract** - we hope that this makes the research approach more explicit. For example:
  - *“The aim of this study was to assess whether service access and presenting concerns differed before and during the pandemic. Sociodemographic characteristics (gender, ethnicity, and deprivation level) were examined to identify disparities in service use.”*

2. One area I thought more could have been said, in the context of the introduction and discussion, was in relation to the voices of young people. I thought the extent to which this digitally based support may be preferable during the context of the pandemic (i.e., with physical distancing etc.), but not outside of it was glossed over and there may be value in citing additional studies which addressed the ‘client’ or stakeholder perspective on digitally delivered support, and how this interlinks with other forms of support (see, e.g., Maddison, C., Wharrad, H., Archard, P. J., & O’Reilly, M. (2024). Exploring young people’s perspectives on digital technology and mental healthcare: pilot study findings. Mental Health Practice, 27(1).) Also, are there ways in which this client voice could be better integrated into this type of CDM study in future?

- Thank you for highlighting the importance of including more detail regarding the voices of young people and the contextual preference for digitally delivered support both during and outside of the pandemic.
- In the **Introduction**, we have included further discussion on youth preferences for digital mental health - both during and outside of the pandemic - with particular emphasis on including qualitative research with young people. See addition on page 2: “*One avenue for improving access to mental health support for CYP is utilising digital mental health support. There are numerous perceived benefits of online mental health interventions, such as anonymity, privacy, and emotional safety due to reduced emotional proximity to the practitioner, as well as increased flexibility, control, and accessibility to treatment (Bambling et al., 2008; Hollis et al., 2017; King et al., 2006). Research exploring youth perspectives suggests that CYP value the potential for interactivity, personalisation, privacy, and sense of anonymity of online support (Dallinger et al., 2022; King et al., 2008; Ludlow et al., 2023). The value of digital support was even more paramount during the Covid-19 pandemic, where digital platforms created opportunity for providing support amongst lockdown restrictions (Chavira et al., 2022; Rauschenberg et al., 2021), especially for CYP with existing vulnerabilities (Mindel et al., 2022). Digital mental health services, therefore, provided an avenue for providing accessible support during the Covid-19 pandemic.”*
- In the **Discussion**, we have highlighted the need for further qualitative investigations to incorporate key stakeholder voices, particularly those of young people, to supplement the present quantitative findings. The need to explore how digitally delivered support can be interlinked with other forms of support has also been raised. Given that we believe that this is not a clinical data mining study, this has not been highlighted. But we hope that this addition highlights the need for future integration of client voice. We have also cited the Mindel et al., (2022) paper which is a qualitative study conducted with youth recruited via Kooth, the same setting as the present paper. Please see:
  - Pp. 16-17: “*Firstly future research can supplement and extend these quantitative findings with a qualitative investigation to explore CYP and key stakeholder voices. Previous research has explored CYP experiences of accessing Kooth during the pandemic and has highlighted its value, particularly for vulnerable CYP (Mindel et al., 2022). However this, alongside qualitative investigations with CYP and mental health professionals (Bell et al., 2024; Maddison et al., 2024) highlight that future research must explore how a digital mental health platform such as Kooth can be integrated alongside face-to-face support (e.g., traditional counselling, school support, etc.) to complement, rather the replace, traditional services. Findings from the present analyses suggest that increased access to the platform was not sustained post-pandemic. Therefore, future qualitative research with CYP and key stakeholders, such as mental health professionals, can explore perspectives on the value of how digital mental health can continue to provide valuable support for CYP in a post-pandemic landscape alongside other forms of support. Indeed, meaningful inclusion of CYP, especially those from marginalised backgrounds or with complex needs, has been highlighted as a key piece in increasing digital mental health uptake and integration (Stiles-Shields et al., 2023).. “*
- P17*: “Specifically analysing factors contributing to ethnic minority access to digital mental health support, alongside qualitative research with CYP, can provide a more nuanced and accurate understanding of this. “*

3. A few more minor issues.

Consider how statistical significance is referred to on pp. 16-17 (see Benjamin, D. J., Berger, J. O., Johannesson, M., Nosek, B. A., Wagenmakers, E. J., Berk, R., ... & Johnson, V. E. (2018). Redefine statistical significance. Nature human behaviour, 2(1), 6-10).

- Thank you for this comment. We have reviewed this citation with interest and agree it highlights an issue of importance. Given the nature of this study we feel that the 0.05 significance level we have chosen is appropriate as the recommendation for more stringent significant levels in the citation above is limited to new scientific discoveries. However, we agree that this raises a valuable point and have added to the limitations section of the paper: *“The use of 0.05 significance levels, while widely used, has been challenged in particular when related to discovery of new effects (Benjamin et al., 2018). Future studies could look to apply more stringent significant levels to strengthen the evidence.”*

At the beginning of the discussion, consider wording used (The paper didn’t itself investigate, but rather the research reported within it did …).

- Thank you highlighting this, we have amended the wording so that this is more accurate. Please see page 12: “*The research within this paper aimed to investigate the…*”

Towards the end of the paper, I’m unsure I agree with the authors that it showcases the value of the service, so much as provides valuable data about access to the service and how it is used. Here, the ‘local’ genesis of the research is perhaps worth acknowledging, i.e., it was undertaken in a single (large) organization and, as much as anything, supports organizational learning.

- Thank you for highlighting this, we agree that this paper provides valuable data about service access and use, as well as opportunity for organisational learning. This has been highlighted in the **Conclusion** on Page 17. “*Overall, this research provides valuable data about service access and usage patterns of a digital platform such as Kooth and how it is used during a time of global need, particularly for vulnerable, harder-to-reach populations. Critically, findings provide an opportunity for organisational learning and research can be leveraged to inform and refine platform design and implementation within similar organisational settings. This is particularly relevant when tailoring such platforms for vulnerable, harder-to-reach populations. For example…*”

Beyond this, I think the point about data availability is perhaps something that the authors want to liaise with the editorial team about, as it appears the authors are working with an anonymized dataset and I’m unsure why this wouldn’t be made available to the public, for example, owing to a freedom of information request. Where Kooth as an organization sits in relation to FoI legislation is, for me, another matter, i.e., they are not a public authority, but they are funded by public monies, so, for me at least, there would be an argument to make the anonymized data available, at least in the interests of transparency for research practice.

- While we agree that there is value in making data publicly available where possible, due to the nature of this data we only make this data available for research studies dedicated to improving the service. Requests to access this data can be made through our research team and we have updated the Data Access Statement to clarify this.

“*The data analyzed in this study is subject to the following licenses/ restrictions: Under Kooth’s privacy policy, data can only be shared with trusted partners for research studies dedicated to improving the service. Requests to access these datasets should be directed to research@kooth.com*”
